# Bio-Decolorization of Synthetic Dyes by a Halophilic Bacterium *Salinivibrio* sp.

**DOI:** 10.3389/fmicb.2020.594011

**Published:** 2020-12-21

**Authors:** Jojy John, Ramadoss Dineshram, Kaveripakam Raman Hemalatha, Magesh Peter Dhassiah, Dharani Gopal, Amit Kumar

**Affiliations:** ^1^Biological Oceanography Division, CSIR-National Institute of Oceanography, Dona Paula, India; ^2^International Research Centre for Climate Change Studies, Sathyabama Institute of Science and Technology, Chennai, India; ^3^Department of Microbiology, Annamalai University, Chidambaram, India; ^4^Department of Marine Biotechnology, National Institute of Ocean Technology, Chennai, India

**Keywords:** phylogenetic analysis, bioremediation, azoreductase, dye decolorization, halophilic bacteria

## Abstract

Synthetic dyes, extensively used in various industries, act as pollutants in the aquatic environment, and pose a significant threat to living beings. In the present study, we assessed the potential of a halophilic bacterium *Salinivibrio kushneri* HTSP isolated from a saltpan for decolorization and bioremediation of synthetic dyes. The genomic assessment of this strain revealed the presence of genes encoding the enzymes involved in decolorization mechanisms including FMN-dependent NADH azoreductase Clade III, which cleave the azo bond of the dye, and the enzymes involved in deamination and isomerization of intermediate compounds. The dye decolorization assay was performed using this bacterial strain on three water-soluble dyes in different concentrations: Coomassie brilliant blue (CBB) G-250 (500–3,000 mg/L), Safranin, and Congo red (50–800 mg/L). Within 48 h, more than 80% of decolorization was observed in all tested concentrations of CBB G-250 and Congo red dyes. The rate of decolorization was the highest for Congo red followed by CBB G-250 and then Safranin. Using UV-Visible spectrometer and Fourier Transform Infrared (FTIR) analysis, peaks were observed in the colored and decolorized solutions. The results indicated a breakdown of dyes upon decolorization, as some peaks were shifted and lost for different vibrations of aromatic rings, aliphatic groups (–CH_2_, –CH_3_) and functional groups (–NH, –SO_3_H, and –SO_3_^−^) in decolorized solutions. This study has shown the potential of *S. kushneri* HTSP to decolorize dyes in higher concentrations at a faster pace than previously reported bacterial strains. Thus, we propose that our isolated strain can be utilized as a potential dye decolorizer and biodegradative for wastewater treatment.

## Introduction

Dyes are organic coloring agents which are extensively used for coloring the products in various industries including textile, paper, cosmetic, and food (Vaghela et al., 2005). These coloring agents are complex aromatic structures which remain stable by resisting the impacts of temperature and other environmental factors (Pandey et al., 2007). Overall, more than thousands of dyes are generated commercially and 7 × 10^5^ metric tons of dyes are produced annually (Amoozegar et al., 2011). Generally, based on their origin, the dyes are classified into natural and synthetic dyes. Natural dyes are obtained directly from plants, microbes, insects, and animals (Shahid et al., 2013). Although natural dyes are eco-friendly and biodegradable, they are unstable (Yamjala et al., 2016). On the other hand, synthetic dyes produced in controlled laboratory conditions show greater stability than natural dyes. Globally, the textile industry utilizes the highest proportion of dyes (Fang et al., 2004), which are often toxic to living beings and difficult to degrade (Fu and Viraraghavan, 2001). Upon coloring the fabrics, 10–15% of used dyes, fibers, and other components generally get discharged into the aquatic ecosystem (Chen et al., 2003). The dyes in the aquatic environment pose a major threat to the inhabitants (Sandhya et al., 2008). The presence of dyes inhibits photosynthetic activity and oxygen solubility at deeper layers of the water body by reducing the light penetration (Saratale et al., 2009). The dyes form a source of aromatic amines upon degradation, which, in turn, are considered as mutagenic, toxic, and carcinogenic, posing threats to living beings (Robinson et al., 2001; Ayed et al., 2011). Considering these detrimental effects of dye on the environment, safe disposal and successful decolorization of dyes becomes the utmost priority.

For dye decolorization and degradation, several methods such as coagulation/adsorption, electrolysis, ozonation, chemical oxidation, and ultrafiltration have generally been used (Zhang et al., 2004; Zhu et al., 2004). However, these methods have limited applicability due to their inefficiency, higher budget, and toxic intermediates (Verma et al., 2003; Zhang et al., 2004). The municipal sewage systems are not adequate to decolorize the water discharge efficiently due to the complex nature of pollutants and residuals from the by-products (Champagne and Ramsay, 2010). Hence, the biological degradation of dyes involving microbes is considered as one of the best methods (O’neill et al., 2000). This has been proven as a cost-effective and eco-friendly method that generates a significantly lesser amount of intermediate toxic compounds (Robinson et al., 2001; Chen et al., 2003). Biological methods include dye degradation by metabolic pathways, absorption, and accumulation by bacteria, fungi, yeast, and algae (Solís et al., 2012). Several studies have reported that bacterial strains possessing genes for azoreductase, laccase, and peroxidase, which act on the amine and aromatic structure of dye, make them suitable candidates for dye degradation (Chen et al., 2003; Babu et al., 2015). Several bacterial strains including Gram-negative and Gram-positive bacteria had been reported to show the dye decolorization ability, for example, *Bacillus subtilus* and *Aeromonas hydrophila* (Wuhrmann et al., 1980); *Proteus mirabilis* and *Pseudomonas* sp. (Saratale et al., 2011); *Shewanella* sp.; and other bacterial consortia (Moosvi et al., 2005).

Major challenges in bacterial dye degradation in the wastewaters include high salt concentration, various metals, and complex nature of wastes. Hence, most of the bacteria, though have shown a promise in the early experiments, fail to work efficiently at a larger scale (Mellado and Ventosa, 2003). In recent years, halophilic bacterial strains have shown promise in dye decolorization (Amoozegar et al., 2011). With the potential to degrade dye, they can grow in a wide range of salinity, temperature, pH, and elevated heavy metal concentrations (Margesin and Schinner, 2001; John et al., 2020).

In the present study, we have assessed the dye degradation potential of a halophilic bacterial strain *Salinivibrio kushneri* HTSP isolated from Marakkanam saltpan, Tamil Nadu, India. This saltpan is characterized by a large seasonal fluctuation of salinity, temperature, dissolved oxygen, and elevated levels of several heavy metals (John et al., 2019, 2020). Previous studies on this halophilic bacterium, *S. kushneri* HTSP (Proteobacteria, Gamma-proteobacteria, Vibrionales, and Vibrionaceae), revealed its ability to grow in a wide salinity range of 15–210 ppt and pH range of 5–10, and it has tolerance to heavy metals including Cu, Zn, Co, Hg, Cr, Pb, and As (John et al., 2019). The analysis of whole-genome sequencing showed the presence of genes conferring resistance/tolerance to heavy metals and UV radiation, as well as those responsible for hydrocarbon degradation, by producing various enzymes, thus indicating the bioremediation potential of this bacterium in various industries and ecosystems (John et al., 2019). Though this species shows potential in dye decolorization, no studies have been attempted to prove this claim. Hence, to address this knowledge gap, we studied bio-decolorization of three dyes belonging to different classes using the *S. kushneri* HTSP strain: (i) Coomassie brilliant blue (CBB) G-250, a triphenylmethane dye commonly used in the textile industry and molecular biology laboratories (Rayaroth et al., 2015; Abbas et al., 2016); (ii) Safranin O, a quinone imine dye commonly used as a biological stain in the laboratories (Drabik et al., 2010; Sabnis, 2010); and (iii) Congo red, an azo dye, a known carcinogen, widely used in the paper, textile and other industries (Babu et al., 2013).

Industrialization is increasing at a faster pace and causing the rise of environmental pollution by producing millions of different chemicals including hydrocarbons, herbicides, pesticides toxic metals, and different dyes as either direct products or by-products (Amoozegar et al., 2011). Synthetic dyes and toxic structures of the dye-containing effluents pose a serious threat to aquatic life forms by causing toxicity to them (Georgiou et al., 2004; Supaka et al., 2004). Biological dye decolorization has been advocated as one of the most suitable methods to treat waste materials before discharge (Stolz, 2001). In this regard, we assessed the suitability of halophilic bacteria *S. kushneri* HTSP isolated from Marakkanam saltpan for decolorization of synthetic dyes. We analyzed the rate of bacterial decolorization of dyes using UV-Vis spectrophotometer and Fourier Transform Infrared (FTIR) spectrometer. We also performed *in silico* analysis to identify genes involved in dye decolorization in the previously reported *S. kushneri* HTSP genome.

## Materials and Methods

### Dyes and Chemicals

Three dyes were used for dye decolorization experiments: Safranin, Congo red, and CBB G-250. All the dyes used in this study were purchased from Sigma-Aldrich (United States). Different concentrations were used for decolorization experiments and the desired concentrations were selected based on preliminary screening from 50 to 5,000 mg/L. For conducting the experiments, 500–3,000 and 50–800 mg/L was selected for CBB G-250, Congo red, and Safranin, respectively. All the dye solutions were prepared in filter-sterilized (0.2 μm) dye decolorization broth for avoiding nutrient loss. The working solutions were prepared using sterilized decolorization media for compensating the nutrient loss.

### Microorganisms and Inoculum Preparation

*Salinivibrio kushneri* HTSP isolated from the Marakkanam salt pan (12.13°02° N; 79.58°12° E) was previously identified through a polyphasic taxonomic approach including morphological, biochemical, and molecular analysis (John et al., 2019). For the present study, the culture was revived from a stock kept in 80% glycerol at −80°C. These were transferred on to a fresh nutrient agar plate prepared in the source seawater (120 ppt). The culture conditions including optimum salinity, temperature, pH, and days of incubation were standardized. The bacterial strain was inoculated under aerobic conditions onto nutrient broth as well as agar with different ranges of salinity, from 15 to 60 ppt, and incubated at 27–37°C for 12 h to 2 days. The required salinity was obtained by mixing the source seawater (200 ppt) with sterilized Milli Q water.

One loopful of overnight-grown culture was inoculated into the decolorization broth (5-g glucose, 2.5-g yeast extract, and 2.5-g NaCl in a final volume of 500 ml) and incubated in a shaker incubator (aerobic) at 37°C for 6 h. After 6 h (log phase of bacterial growth), the cells were harvested by a quick centrifuge at 3,000 rpm for 5 min. The bacterial cells were resuspended in 0.8% NaCl, and the optical density at 600 nm was adjusted to ~0.6–0.8 (6 × 10^8^ cells/ml). The cell suspensions were used for further analysis.

### Decolorization Experiment

Two hundred microliters of the bacterial cell suspension were inoculated onto different concentrations of different dyes (for 20 ml) and the tubes were incubated at 37°C for 48 h against a negative control, which contained only the dye in respective concentrations without the inoculum. The solution was withdrawn at an interval of 4 h for CBB G-250 and 6 h for Congo red and Safranin. The absorbance of the decolorized media solution (blank) was measured at 580 nm (CBB G-250), 490 nm (Congo red), and 530 nm (Safranin), respectively, using UV-Vis spectrophotometer against a blank of decolorization media without dye. The solution was centrifuged at 12,000 rpm for 1 min before taking the absorbance for removing any suspended precipitates. All the experiments were performed in triplicates. The trial experiment was also conducted with a higher volume (250 ml) and the same result was obtained. For the qualitative and quantitative analysis, the experiment was performed in 20 ml volume.

Decolorization percentage was calculated using the formula:

$$\mathrm{Decolorization}\;\left(\%\right)=\left[\frac{\mathrm{Initial absorbance}-\mathrm{final absorbance}}{\mathrm{Initial absorbance}}\right]*100$$

### Biodegradation Assay

The dye degradation was further confirmed through spectral analysis of UV-Vis spectroscopy and FTIR spectroscopy.

### UV-Visible Spectroscopy

For the UV-Vis spectral analysis of dye decolorization, the decolorized solution was scanned (200–1,000 nm) against a dye control and the peaks were cross-matched using a spectrophotometer (Shimadzu, UV-1800). The highest concentration of dye which was completely decolorized was chosen for this analysis against a dye control. The peaks obtained before and after decolorization were analyzed (Ali et al., 2009; Ayed et al., 2011).

### Fourier Transform Infrared Spectroscopy

The functional groups of the degraded dyes were analyzed with FTIR spectroscopy. The biodegraded dye was collected after the experiment and the bacterial suspension was removed by centrifugation at 5,000 rpm for 5 min. The resulting supernatant solution was lyophilized and used for FTIR analysis. The highest concentration at which the dyes were completely decolorized was used as a test sample for FTIR analysis and the dyes in powder form were used as control. The mid-IR spectra of degraded dyes were obtained in the FTIR spectrophotometer by Shimadzu, IR Infinity 1. The degraded dye sample was prepared by mixing 1 weight % of dye with 99 weight % of KBr and the mixture was ground well to make a paste of uniform consistency. The sample matrix was loaded in a 7-mm Pellet Die of 7-mm Disc Holder Mount and pressed in 2 T Mini-Pellet Press (Specac Ltd.). The sample mixture was then analyzed using the instrument by mounting the sample on the sample holder using the ring holder and the transmittance scanned from the range of 4,000–400 cm^−1^ with a resolution of 1 cm^−1^ set for 20 scans per min (Babu et al., 2013). The obtained peaks were compared with the published reference dye peaks (Sigma-Aldrich, n.d.; Sun et al., 2013; Maity et al., 2015; Sahu and Patel, 2015; Asses et al., 2018).

### *In silico* Analysis of *Salinivibrio kushneri* HTSP Dye-Decolorizing

The genome of *S. kushneri* HTSP was retrieved from the NCBI genome database (John et al., 2019, NCBI genome accession no. PXUD00000000) for analyzing dye-decolorizing genes. The genome was subjected to automatic annotation on the Rapid Annotations using Subsystems Technology webserver (Overbeek et al., 2014). The annotated genome was visually searched to identify the target genes, and a BLAST search was performed using the annotated genome against well-studied genes.

Azoreductase Clade III, a well-known decolorizing enzyme, was identified in the genome and compared with other bacterial species through phylogenetic analysis. The phylogenetic tree was constructed based on an amino acid sequence from the best BLASTp hits along with previously reported sequences (Kumaran et al., 2020). Pairwise and multiple sequence alignment was performed using the CLUSTAL W program and phylogeny was constructed using the Neighbor-Joining (NJ) method in MEGA (v 7.0, Kumar et al., 2016). Multispecies nitroreductase family protein of *Bacilli* (NCBI GenBank accession: WP_002358386) was used as an outgroup in the phylogenetic tree.

### Statistical Analysis

All the data from dye decolorization assays were tested for statistical significance by comparing the mean of different test conditions using One-way ANOVA with the Tukey Scheffe alpha multiple comparison test. The data were checked for the normality by visual inspection and homogeneity of variance by Levene’s test. The data were considered significant if *p* < 0.05. The statistical analysis was performed using SPSS [V. 22, IBM SPSS Statistics for Windows, Armonk, NY (SPSS, 2013)].

## Results and Discussion

### *In silico* Analysis for Dye Decolorization Genes

A total of 12 genes involved in the dye decolorization pathway were identified. These include genes encoding FMN-dependent NADH azoreductase Clade III, hydroxymuconate delta isomerase, maleylacetoacetate isomerase, cytidine deaminase, cytosine deaminase, ornithine cyclodeaminase, deoxycytidine triphosphate deaminase, tRNA-specific adenosine-34 deaminase, diamino hydroxyl phosphoribosyl amino pyrimidine deaminase, glucosamine-6-phosphate deaminase, adenosine deaminase, and porphobilinogen deaminase.

**Figure 1:**
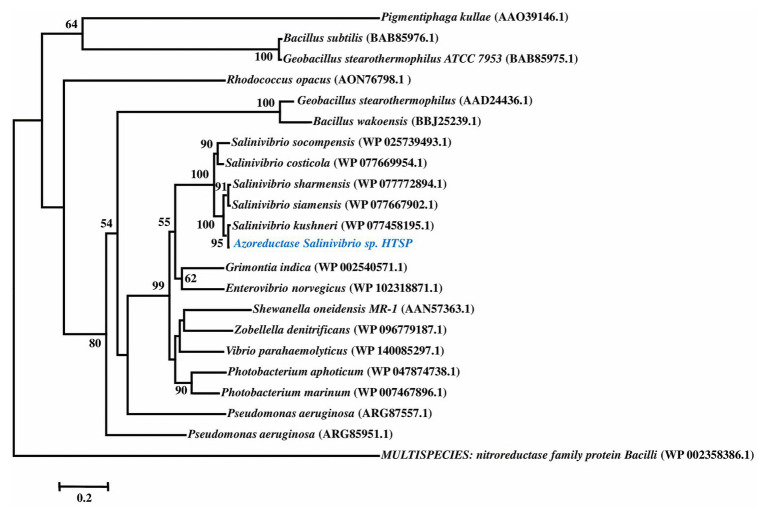
Phylogenetic tree of azoreductase based on amino acid sequences. Amino acid sequences for nitroreductase is used as an outgroup.

To ascertain the identity of the FMN-dependent NADPH azoreductase Clade III, the first enzyme in the decolorizing pathway (Dave et al., 2015), the amino acid sequence of the gene was compared with previously reported sequences through BLAST and NJ-based phylogeny. The phylogenetic reconstruction revealed that azoreductase of *S. kushneri* HTSP forms a separate clade supported by high bootstrap confidence (Figure 1).

**Figure 2:**
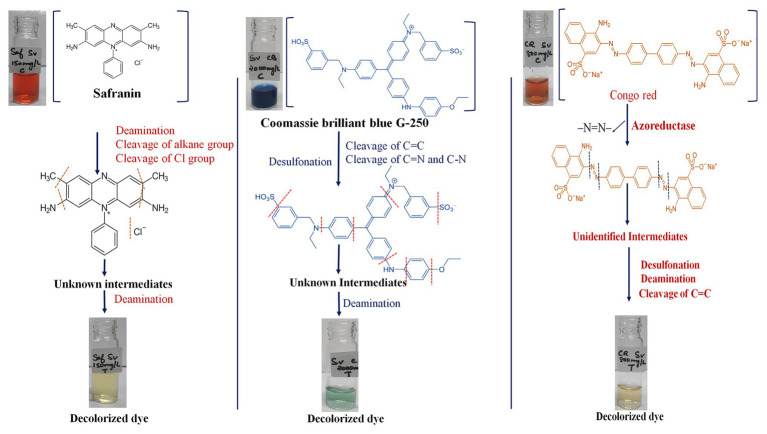
Predicted mechanism of dye decolorization by *Salinivibrio kushneri* HTSP based on Fourier Transform Infrared (FTIR) peaks and functions of the genes identified. Functional group and bond cleavages are identified through the FTIR peaks.

The genomic assessment of this strain revealed the presence of genes involved in various mechanisms of dye decolorization, for example, the FMN-dependent NADH azoreductase is involved in cleaving the azo bonds of the dye and converting the dye into aromatic amines (Dave et al., 2015). The resultant aromatic amines and other functional groups would be further cleaved or reduced by genes involved in deamination and isomerization processes. Based on the genes found in the genome, we have predicted a dye decolorization pathway (Figure 2). However further analysis like NMR; GC/LC-MS is needed for the confirmation of formed intermediates. AZO reductase, a primary enzyme in the decolorization process, was compared with other known sequences from different bacterial strains. Though the sequences from the genus *Salinivibrio* including *S. kushneri* HTSP formed a separate clade in the phylogenetic tree, it was comparatively closer to clade III azoreductase than other classes (I, II, and IV) of azoreductases (Suzuki, 2019; Kumaran et al., 2020). Class III azoreductase is a flavin-dependent enzyme with a significant difference in substrate specificities (Bafana and Chakrabarti, 2008; Misal and Gawai, 2018). In general, azo, nitro compounds, and quinone are a better substrate for class III azoreductase (Liu et al., 2008; Suzuki, 2019) such as Congo red (Yang et al., 2011; Gao et al., 2015), dyes with methyl groups (Eslami et al., 2016). Hence, it can be assumed that *S. kushneri* HTSP uses azoreductase to decolorize Congo red, Safranin (a dye with methyl group), and CBB G-250, indicating its potential for dye decolorization. The ability of different bacterial strains to decolorize different classes of dyes through the action of azoreductase has been reported earlier (Eichlerová et al., 2006; Amoozegar et al., 2011). For example, *Halomonas elongate*, a halophilic bacterium, is reported to decolorize mono and di azo dyes including methyl red, remazol black, and sulphonyl blue TLE (Eslami et al., 2016; Cao et al., 2017). Further, to ascertain our claim based on genomic analysis that the isolated strain can achieve dye decolorization, we performed decolorization assays on three dyes Congo red, Safranin, and CBB G-250 and analyzed using analytical methods of UV-Vis spectrophotometer and FTIR spectrometer, which is considered a gold standard for decolorization studies (Chen et al., 2008).

### Dye Decolorization by *Salinivibrio kushneri* HTSP

**Figure 3:**
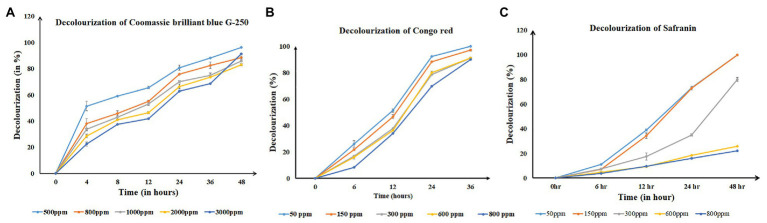
Time-series graph denoting the decolorizing percentage of all three dyes at regular time intervals for *S. kushneri* HTSP, where, **(A)** is Coomassie brilliant blue G-250 (CBB), **(B)** is Congo red, and **(C)** is Safranin.

The bacterial strain showed a luxuriant growth on a 60 ppt solid plate after overnight incubation at room temperature (~29°C) with a pH of 7.4, and in the broth, it took 6 h to reach an optical density of 0.8–1 at 600 nm. Hence, decolorization assays were performed in 60 ppt salinity conditions. Single-factor analysis on ANOVA after complete decolorization of each dye at same time point showed the rate of decolorization between the different concentrations of dyes was statistically significant (*p* < 0.05). *Salinivibrio kushneri* HTSP significantly decolorized CBB G-250 dye for all concentrations within 48 h (Figure 3A). The percentage of decolorization was slow in the initial period of incubation and by 48 h almost 90% of the dye at all tested concentrations were completely decolorized. Within 48 h, 96 ± 0.00, 88 ± 0.00, 85 ± 0.00, 82 ± 0.02, and 73 ± 0.00% dye was decolorized in 500, 800, 1,000, 2,000, and 3,000 mg/L dye, respectively (Figure 3A).

For Congo red dye decolorization, a significant color reduction was observed within 36 h of incubation. At 24 h, 92 ± 0.00% dye was decolorized in 50 ppm and for 150, 300, 600, and 800 ppm the decolorization percentage was found to be 88 ± 0.00, 78 ± 0.01, 80 ± 0.02, and 69 ± 0.00%, respectively. After 36 h, more than 90% of dye was decolorized in all concentrations (Figure 3B).

*Salinivibrio kushneri* HTSP decolorized Safranin completely in the lower concentration of 50 and 150 mg/L within 48 h. However, with increasing dye concentration, the rate of decolorization decreased. A low level of decolorization, such as 79 ± 0.02% for 300 mg/L, 25 ± 0.00% for 600 mg/L, and 22 ± 0.00 for 800 mg/L was obtained after 48 h (Figure 3C).

### Characterization of Samples After Degradation

**Figure 4:**
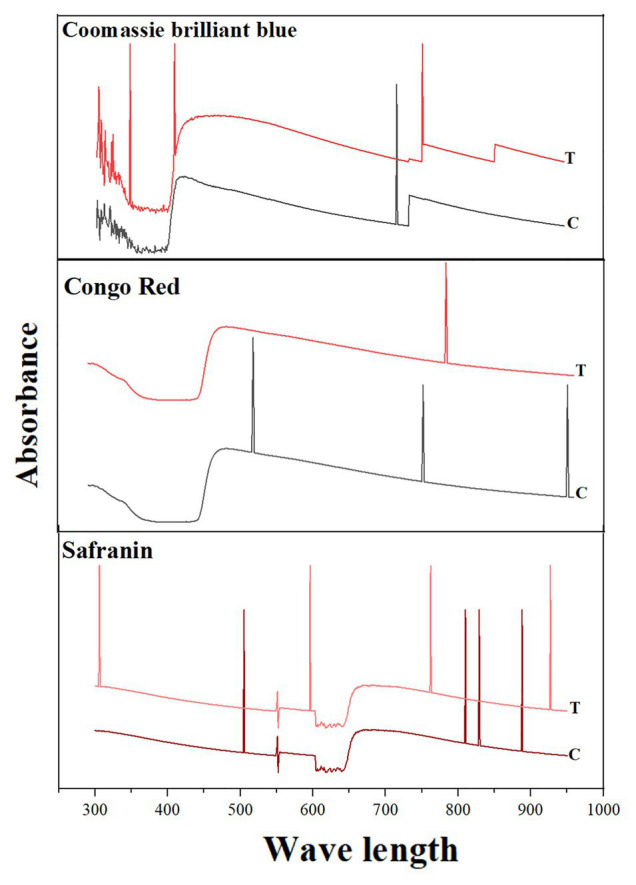
Spectrophotometric analysis of dye decolorization by *S. kushneri* HTSP. Here, Control indicates dye before decolorization and test indicates dye after decolorization. CBB for CBB G-250.

Shifts in the peaks were observed under UV-Vis spectroscopy between the test decolorized samples and the control. For CBB G-250, only one peak was observed in the control (at 585 nm) and three peaks were observed in the test decolorized sample. For Congo red, three different peaks were observed in the control, while only one peak after decolorization. For Safranin, four peaks were observed in both the control and the decolorized sample. However, the absorption wavelengths were different before and after decolorization: Peaks were observed at 888, 829, 810, and 505 nm before decolorization and 927, 732, 596, and 306 nm after decolorization (Figure 4).

**Figure 5:**
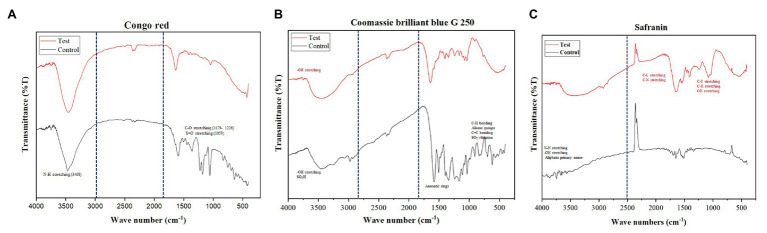
Functional group analysis of all dyes before and after decolorization. Control indicates dye before decolorization and test indicates dye after decolorization. The important functional groups, aromatic rings, and aliphatic groups are highlighted in the control and in the test. Where, **(A)** is Congo red, **(B)** is CBB G 250, and **(C)** is Safranin.

Further, various peaks were obtained in FTIR analysis of all three dyes corresponding to different vibrations of aromatic rings, aliphatic groups (–CH_2_, –CH_3_), and functional groups (–NH, –SO_3_H, and –SO_3_^−^). There were considerable variations observed in the functional groups before and after dye decolorization. For Congo red, 12 absorption peaks were observed before decolorization; however, no relevant peak was observed after decolorization (Figure 5A). For CBB G-250, 33 peaks were observed in the colored solution, while the number of peaks reduced to 30 after decolorization. The peaks spanned in the range of 422–3,200 nm wavelength with a majority between 1,000 and 2,000 nm. However, peaks were not the same between colorized and decolorized solution, except for one at 454 nm (Figure 5B). For Safranin, 25 peaks in color solution and 24 peaks in decolorized solutions were observed. However, colored solution peaks were in the range of 3,400–4,000 nm, and decolorized solution peaks in the range of 600–3,600 nm (Figure 5C). We used smoothened data for plotting the graph.

The functional group analysis using FTIR and peak analysis using UV-Vis spectroscopy before and after 36 h of decolorization of CBB G-250 (2,000 mg/L), Safranin (150 mg/L), and Congo red (800 mg/L) supported our predicted mechanism shown in Figure 2. In general, the peaks which are responsible for several different bonds and functional groups cleavage were identified and matched. The major peaks corresponded with cleavage of N=N bonds, cleavage of aromatic rings, OH releasing, conversion of SO^3−^ to –OH groups, cleavage of C=C and C=H bonding, and cleavage of alkane group. On the contrary, after decolorization, the majority of the peaks were identified as hydroxy groups (–OH; Maity et al., 2015).

There were no significant peaks were observed after decolorization for Congo red. For Congo red dye (Figure 5A), observed peaks were identified as N–H stretching (3,468 cm^−1^), C–O stretching (1,226–1,179 cm^−1^), S=O stretching (1,059 cm^−1^), and several unknown peaks found in the control sample were absent in the test conditions (Babu et al., 2013; Sun et al., 2013; Asses et al., 2018). For CBB G-250, the peaks in the range of 1,397–1,585 nm that corresponded with aromatic rings observed in the colored solution were mostly absent in the decolorized solution. Sulfur dioxide vibration (1,000–1,200 cm^−1^), C=C (985–618 cm^−1^) bending, CH bending (985–618 cm^−1^), and alkane groups (2,291–2,974 cm^−1^) were observed in the colored solution but not in the decolorized solution, as shown in Figure 5B (Maity et al., 2015; Paz et al., 2017). For Safranin, the absorbance peaks observed were mainly aliphatic primary amine N–H stretching (3,000–3,560 cm^−1^) and –OH stretching in the range of 3,589–4,000 nm in the control sample. However, in the decolorized test sample, the absorbance peaks varied: C–L stretching at 625 nm, C–N stretching 1,042–1,626 nm, C–C stretching 1,626 nm, C–H stretching 2,853–3,243 nm, and –OH stretching 3,283–3,564 nm (Babu et al., 2015; Sahu and Patel, 2015). The shift in the peaks suggests a breakdown of functional groups that may have changed the original structure of the dyes in the decolorized test samples (Chen et al., 2008; Ali et al., 2009).

Our results clearly indicate that *S. kushneri* HTSP could decolorize up to 3,000 mg/L CBB G-250; 300 mg/L for Safranin; and 800 mg/L for Congo red, which is significantly a higher concentration than previously reported. For example, Paz et al. (2017) showed that *Bacillus aryabhattai* could decolorize CBB G-250 up to 150 mg/L; Babu et al. (2013) showed that *Dietzia* sp. decolorized Congo red up to 100 mg/L. In addition, the time for decolorization was also found to be significantly less for *S. kushneri* HTSP than previously reported. Our results have shown that *S. kushneri* HTSP could decolorize a higher concentration of dyes at even a faster rate than previously reported halophilic bacteria such as *Shewanella putrefacians* and *Halomonas* sp. (Amoozegar et al., 2011; Gao et al., 2015). Previous studies on other bacteria also have reported a higher average time to decolorize the dyes used in this study even in comparatively lower concentrations (Babu et al., 2013; Paz et al., 2017).

## Conclusion

Earlier studies have shown that *S. kushneri* HTSP has proven capacity to grow in a wide range of salinity, as well as the ability to tolerate/resist heavy metals and UV radiation (John et al., 2019), and now we report that this strain has capability to decolorize dyes in the higher concentration at a relatively faster rate. Thus, we argue that it can be potentially utilized for dye decolorization in wastewater treatment. However, further studies including cytotoxicity assays are to be conducted before using this strain at the industrial scales.

## Data Availability Statement

The data used in current study can be found in NCBI repository and all the accession numbers are provided in Materials and Methods section.

## Author Contributions

JJ and RD designed the research. JJ performed the decolourization experiments with KH. RD performed FTIR analysis with the help of MD and DG. JJ, RD, and AK analyzed the data and drafted the final version of the manuscript to be published. All authors contributed to the article and approved the submitted version.

### Conflict of Interest

The authors declare that the research was conducted in the absence of any commercial or financial relationships that could be construed as a potential conflict of interest.
